# “Massage” in Sprains

**Published:** 1875-04

**Authors:** 


"Massage" in Sprains.—The Paris correspondent of the *Irish Hospital Gazette* says:

M. Broca does not believe in the efficacy of absolute rest in sprains, and attaches greater importance to shampooing than is generally accorded to it by surgeons. Its omission in ordinary practice, he said, was much to be regretted, and it would in some measure account for the success of "rebouters," (bone-setters) who infest the provinces and employ this mode of treatment to a great extent, almost to the entire exclusion of rest, so much insisted on by regular surgeons, not only to the prejudice of the patient's health, but of his purse, and, in the case of a workman, perhaps of his livelihood for a time. M. Broca expressed his surprise that the subject is so lightly treated in some classical works, while others do not even mention it as a remedy for sprains or any other malady; he therefore took occasion to explain what shampooing was, and its mode of action in the treatment of sprains, etc., as follows: "Primary Shampooing," he stated, consisted of pressing or kneeding the swollen tissues with the fingers; then of alternately flexing and extending the joints affected. By this pressure and forced motion the extravasated liquids are dispersed into the subjacent cellular tissue. After the first shampooing the pain and swelling return, but on the second day, when the operation is repeated, its effects last much longer, the pain is diminished, and after a few days, during which the operation is regularly practised, the pain and œdema disappear completely. "Secondary Shampooing" is applicable to cases that had not been treated, or imperfectly so, in the first instance, and in which the pain, swelling, and inability to move have persisted. In such a case he would begin with gentle frictions, which are to be gradually increased, and to be applied to the most painful parts.

The counter-indications against this mode of treatment consist of acute inflammation of the parts; as in such a case the operation of shampooing would not only be intolerable, but would increase the inflammation. In all cases of sprain the utmost care and attention should be paid with a view of forming a diagnosis, as it would be unpardonable in any surgeon shampooing a fractured limb, a practice not infrequent among quacks and bonesetters. In case of doubt, better treat the patient upon ordinary principles than to resort to the cruel and unscientific method of shampooing under such circumstances. M. Broca then described the process of the operation. After each sitting he applies a roller steeped in Goulard or some other resolvent lotion, and enjoins rest, absolute or otherwise, according to the nature of the case.

